# Fungi Recorded on Folivorous Lepidoptera: High Diversity Despite Moderate Prevalence

**DOI:** 10.3390/jof7010025

**Published:** 2021-01-05

**Authors:** Robin Gielen, Hendrik Meister, Toomas Tammaru, Kadri Põldmaa

**Affiliations:** 1 Entomology Unit, Department of Zoology, Institute of Ecology and Earth Sciences, Faculty of Science and Technology, University of Tartu, Vanemuise 46, Tartu 51014, Estonia; hendrik.meister@gmail.com (H.M.); toomas.tammaru@ut.ee (T.T.); 2 Mycology Unit, Department of Botany, Institute of Ecology and Earth Sciences, Faculty of Science and Technology, University of Tartu, Ravila 14a, Tartu 50411, Estonia; hypomyc@ut.ee

**Keywords:** Geometridae, Hypocreales, mortality, moth, larva, pupa, entomopathogen

## Abstract

The knowledge about the diversity and ecological role of entomopathogenic fungi is primarily based on agroecosystems whereas information derived from natural insect populations is much more limited. To contribute to filling this gap, we recorded the prevalence of fungal infections in laboratory rearing experiments with five species of Lepidoptera, and in a field rearing experiment including one of these moths. The diversity of detected fungi was found to be high; we isolated 25 species of fungi from insects that had died in the course of these experiments. Six species belonged to the family Cordycipitaceae known to include unambiguous insect pathogens. The trophic niche of the representatives of other taxa is less clear and requires further studies. Regarding the most abundant species, *Cordyceps farinosa,* in which this question could be addressed, there was no indication of specialization on particular insect hosts, whereas several of the less common species may have been recorded from lepidopteran hosts for the first time. Across the subsets of the data, the prevalence of fungal infections generally remained below 5%. Our results are thus consistent with the idea that entomopathogenic fungi are always present in insect populations but rarely reach epizootic levels. The detected species richness shows that much is to be gained from mapping the diversity of fungal species associated with folivorous insects in natural populations.

## 1. Introduction

Focusing on the interactions of insects with plants and microbes has been considered the key to better understand various mechanisms behind ecosystem functioning [1]. The significance of insects is modulated by their abundance and the diversity of biotic interactions, most prominently of those with various natural enemies. As insects cause great losses in forestry and agriculture, the understanding of the biology of their natural enemies is of high importance also in the applied context [2,3,4]. Thus far, parasitoids and vertebrates have received most research effort in the context of ecological studies on insects [5,6]. Naturally, pathogens have also not escaped attention as antagonists of insects [7,8,9]; among those, viruses have had a central role in ecological studies [10]. Moreover, from the late 1990s, there has been an exponential growth in studies focusing on the use of fungi as biocontrol agents [2,11]. However, the knowledge about the role of entomopathogenic fungi in natural settings is still scarce, despite the almost two centuries long awareness about these organisms in the scientific community [12].

To contribute to filling this gap, we recorded the diversity and prevalence of lethal fungal pathogens in laboratory rearing of lepidopteran larvae that represented offspring of field-collected females, fed with field-collected host plants. The assemblage of fungal pathogens in the laboratory was compared to that recorded in a field experiment in which moth larvae were reared in a seminatural setting. The focus was set on plant- and air-borne fungal infections relevant for folivorous larvae, with the interactions of soil-dwelling pupae [2,3] remaining beyond the scope of the present paper. We discuss the patterns of diversity and abundance of insect-associated fungi on lepidopteran hosts, and report taxa not recorded on this group of insects previously.

## 2. Materials and Methods

### 2.1. Laboratory Rearing

Data on fungal pathogens present in laboratory rearing of Lepidoptera were obtained as a by-product of ecological experiments performed at the University of Tartu, Estonia, in 2014-2017. Four lepidopteran species—*Ematurga atomaria* L., *Cabera pusaria* L., *Hypomecis punctinalis* Scopoli (Geometridae), and *Orthosia gothica* L. (Noctuidae)—were subjected to identical experimental design ([13,14,15], and unpublished). Specifically, in order to record growth rates, developmental periods, and final weights, we reared the larvae from eggs to pupae singly in 50 mL plastic vials at different temperatures and weighed them periodically. Within each combination of year and species, the insects were reared simultaneously under common garden design, while the timing of experiments with different species did not coincide due to natural phenological differences of the insects. The data on the fifth species, *Chiasmia clathrata* L. (Geometridae), were obtained from a technically similar study in which growth parameters of selection lines were compared (Välimäki et al., unpublished). The larvae were fed with leaves of host plants collected in the field in Tartu, or surroundings of the town. The larvae of *E. atomaria* were fed on *Trifolium repens* L. (Fabaceae), *Vaccinium myrtillus* L. (Ericaceae) and *Salix alba* L. (Salicaceae); *C. pusaria* on *Alnus glutinosa* (L.) Gaertn. (Betulaceae); *H. punctinalis* on *Betula pendula* Roth. (Betulaceae), *Tilia cordata* Mill. (Tiliaceae), and *Quercus robur* L. (Fagaceae); *O. gothica* on *B. pendula*; and *C. clathrata* on *Lathyrus pratensis* L. (Fabaceae).

The larvae were allowed to pupate in moist *Sphagnum* moss, known for its antiseptic properties. This should have minimized the insects’ risk of being infected during the pupal period, allowing us to focus on infections acquired during the larval stage. The pupae were kept overwinter in thermoregulated chambers at about 0 °C. In spring, adult moths were allowed to eclose at room temperature. The pupae that failed to eclose and eventually died were inspected for visual signs of fungal infection.

### 2.2. Field Experiment

To record the community of insect-associated fungi in near natural conditions, we reared larvae of *C. pusaria* on living wild host plants in mixed forest fragments in the surroundings of Tartu (between 58°26′ N, 26°30′ E and 58°24′ N, 26°39′ E), Estonia, in the course of 2 years. Newly hatched larvae were enclosed in 50 × 30 cm^2^ polyester bags (*N* = 81), in which they remained until pupation. Ten larvae per bag were placed on 3 different host plants of the moth—grey alder (*Alnus incana* (L.) Moench), silver birch (*Betula pendula* Roth.), and downy birch (*B. pubescens* Ehrh.)—in the first half of July 2016 and 2017 and were checked weekly for pupation. After pupation, the insects were placed individually into Empera 124 N polystyrene vials with sterilized peat moss (*Sphagnum* sp.). The moss was sterilized by keeping it at 100 °C for 4 h. Vials were sterilized with 10% NaOCl. Pupae overwintered in thermoregulated chambers at 2 °C for 3 months. In January, adult moths were allowed to eclose at 24 °C and 12:12 h of light/darkness cycle. Insects that died during the pupal period were inspected for visual signs of fungal infection.

### 2.3. Identification of Fungi and Their Host Ranges

To identify the fungi and preserve these as pure living cultures, we inoculated visible fungal material (only anamorphs were encountered) to Petri dishes with 2% malt extract agar (Oxoid, Cambridge, UK) supplemented with antibiotics (1% of streptomycin and tetracycline). After a few weeks of growth in culture, the fungi were identified on the basis of morphological traits using keys provided by Domsch et al. [16], Samson et al. [12], and Seifert et al. [17]. A culture isolate representing each morphotype was subjected to DNA extraction to confirm the identification.

The procedures of growing the mycelium, extracting DNA, conducting PCR, and sequencing followed the protocols described by Põldmaa et al. [18]. Ribosomal DNA full ITS and partial LSU sequences were obtained from 91 fungal isolates. The sequences along with their metadata were uploaded in PlutoF, a data management and publishing platform [19], and made available via UNITE database [20]. The ITS rDNA sequences were incorporated in the UNITE species hypotheses (SH), which served as the basis for species identification, by choosing an appropriate distance threshold value in each case (Table 1). The Basic Local Alignment Search Tool (blastn) at National Centre for Biotechnology Information [21] was used to check for similar sequences not yet incorporated in the UNITE database.

All pupae infected with a fungus were deposited at the TU fungarium (accession numbers TU133001-133196) and representative isolates at the TFC culture collection (TFC202234-202344) in the Natural History Museum and Botanical Garden, University of Tartu.

## 3. Results

### 3.1. Fungi from the Laboratory Experiments and Their Prevalence

Data on 2978 lepidopteran pupae were obtained from the laboratory rearing experiments (Table 2). Fungi were detected from 82 pupae (2.8%) and identified as representing 23 species from 10 families and 6 orders (Table 1). The prevalence of fungal infections differed among the 3 years but remained below 10% (Table 2, note the small sample sizes of *C. pusaria* and *H. punctinalis* in 2016, associated with atypically high prevalence). The majority of the fungi belonged to Cordycipitaceae (59 pupae infected), followed by Aspergillaceae (8), Nectriaceae (7), Mortierellaceae (4), Hypocreaceae (4), Tilachlidiaceae (3), Clavicipitaceae (2), Saccotechiaceae (2), Umbelopsidaceae (1), and Cladosporiaceae (1).

On the basis of previous knowledge [12], all of the Cordycipitaceae (six species, overall prevalence 2.2%) were considered to unambiguously represent entomopathogens infecting living hosts. The most abundant of such species, *Cordyceps farinosa*, was found in total on 43 pupae of 3 moth species out of 5. While present each year, it was the most prevalent entomopathogen in 2015 and 2016. Species from other families should be considered potential (opportunistic) pathogens of Lepidoptera as most of them belong to large genera including saprotrophs and pathogens of various hosts, with some representatives occasionally found also on insects [23,24,25]. There are exceptions from this general scheme, however. In particular, while several species of Clavicipitaceae are known as entomopathogens, the genus *Metapochonia* has been mainly found on nematodes. The genus is represented here by the nematode and rotifer pathogen *Metapochonia bulbillosa* [26], whereas the respective UNITE SH (Table 1) also includes a few sequences obtained from Coleoptera. Moreover, *Tilachlidium brachiatum* has been known to grow only on decaying fungi (K. Põldmaa, personal observation). In addition, our data may include the first records on Lepidoptera/insects for some species from the genera *Mariannaea*, *Mortierella*, *Simplicillium*, *Trichoderma,* and *Umbelopsis*. However, the respective host associations need further investigation as species concepts in these groups are changing as a consequence of advances in molecular systematics.

Lepidopteran species differed in the prevalence of infection—*O. gothica* had the lowest rate (0.4%), while *E. atomaria* and *C. pusaria* had the highest (5% and 3.3%, respectively). There was also a difference between the 3 years of study, showing a trend of increase in the fungal prevalence (Table 2). However, given the somewhat non-systematic character of the data (rearing experiments were not designed to study the prevalence of infections), we refrain here from presenting formal statistical analyses.

### 3.2. Field Experiment

Of the 868 first instar *C. pusaria* caterpillars released for the experiment, 296 (34.1%, Table 3) insects reached pupation. Fungi were detected on 87 out of the 191 dead pupae (45.5%). However, if we consider only Cordycipitaceae (3 species on 37 pupae), the average prevalence of fungal infections drops to 17.3% (2.2% in 2016 and 24% in 2017). The prevalence of fungi was thus considerably higher in the field compared to the lab rearings. The detected taxa (10 species, Table 1) overlapped with those that were identified in the laboratory rearing, except for two species of Mucorales, known as ubiquitous saprotrophs (but see also [24]). The 19 pupae, infected by a member of Cordycipitaceae plus another fungus, suggest that the latter may represent saprotrophs exploiting the already dead moth tissue.

## 4. Discussion

Our results suggest that potentially entomopathogenic fungi are invariably present in insect populations. Fungal infections were recorded in all subsets of our laboratory rearing data (host species * year), with just one exception (*C. pusaria* in 2014, but note the low samples size, Table 2). The detected diversity of fungi was notably high. Altogether, 25 species of fungi from 7 orders were isolated from the laboratory and the field experiments, with two-thirds (16 species) belonging to the Hypocreales (Table 1). Six of the collected fungi could not be unambiguously affiliated to a described species on the basis of their ITS DNA sequences (Table 1), and UNITE species hypotheses with respective DOI codes [22] are used for communicating on these. This might have been due to the possibility that cryptic undescribed species were involved, or that voucher specimens of known species have not yet been sequenced, or the inapplicability of the ITS region for discriminating sibling species. Greatest fungal diversity was found on *Ematurga atomaria* (17 spp.), followed by *Cabera pusaria* (11), *Hypomecis punctinalis* (6), *Chiasmia clathrata* (2), and *Orthosia gothica* (2). However, the numbers of species recorded for each host are well consistent with species-specific sample sizes (Table 2) so that these figures should not be interpreted as an indication of differences in the community of fungi associated with different moth species.

Among the unambiguously entomopathogenic fungi, here defined as members of Cordycipitaceae, we identified two abundant (*Akanthomyces muscarius* and *Cordyceps farinosa*) and five scarce species (Table 1). In addition, members of the families Hypocreaceae, Nectriaceae, Aspergillaceae, and Mucoraceae were frequently found growing on dead insect pupae. These fungi were especially common in the field experiment, often accompanying a species of the Cordycipitaceae. Therefore, we may consider such fungi to primarily take advantage of pupae killed by other pathogens, but it cannot by any means be excluded that some of these may still possess thus far unrecognized opportunistic abilities to cause the death of the insects (see [24]). Further studies are needed to establish the nutritional strategies of fungi that are repeatedly found on dead insects.

Our study focused on fungal infections of folivorous larvae, which can only be brought about by plant- and air-borne propagules. In particular, the hosts were not in contact with soil and leaf litter, which are the environments considered to constitute reservoirs for entomopathogenic fungi [2,4]. This may explain why our samples did not include some well-known and common entomopathogens, such as *Beauveria* and *Metarhizium* spp., and suggests that the full spectrum of the fungi associated with natural populations of the studied insects may be considerably broader than recorded in the present study. Several of the presumable saprotrophs/potential entomopathogens, for which we have identified no or just very few previous records on insects, have been reported from plants, soil, or also from air and water. The fact that such fungi were more common on pupae from the field than from the lab experiment suggests that prolonged exposure to the natural environment favors the deposition of different fungi on insects and/or their host plants (but see also [2]).

Our laboratory-derived data do not suggest any strong specialization of the fungi to particular host species. However, the just moderate amount of data available did not allow us to perform any formal analyses of specialization patterns. Indeed, 9 of the 13 fungal species that were found only on one host were represented by just a single observation. Nevertheless, it should be noted that the most abundant species—*Cordyceps farinosa* and *Akanthomyces muscarius*—were both found as readily infecting all the three most numerous hosts (*C. pusaria*, *E. atomaria*, and *H. punctinalis*), with no evidence of preferring one species over the other.

The overall prevalence of fungal infections in our laboratory rearings varied among different subsets of the data from 0 to 6% (excluding subsamples with less than 100 pupae). This value was 13.3 and 55.5% for the 2 years of the field experiment (0 to 6% vs. 2.2 and 24%, if to consider Cordycipitaceae only), indicating 10 times higher incidence of fungal infections than in the laboratory rearings. These values of prevalence can alone be interpreted as evidence of a non-negligible role of the insect–fungus interactions in the ecology of studied moths.

The lab-based estimates can underestimate the prevalence of entomopathogens in nature as the insects in the laboratory culture should be less exposed to various potential sources of infection than in the field, e.g., soil or infected insects [23,27]. Alternatively, laboratory mass rearings of insects might be prone to disease outbreaks, leading to higher prevalence values in the lab compared to the field. This appears not to have been the case, as epizootic levels were not reached in any of the subsets of our data. Additionally, the diversity of fungal pathogens recorded in the lab, as well as their similarity with field collections, provides evidence against outbreak of a particular fungus in our experimental facilities. The observation that fungal pathogens are always present at low frequencies is well consistent with the decades-long experience of insect rearing by some of the authors (but see [28]). Such a pattern might indicate that the presence of fungal conidia is not a limiting determinant of the prevalence of fungal diseases (see also [29]) but instead, the condition of the host may be decisive—only the weakest individuals are unable to resist the infection [4,30].

Currently, ecological knowledge about entomopathogenic fungi is primarily based on studying a few well-known species of fungi and isolating entomopathogens mainly from soil rather than describing complete fungal communities of particular insect species [3]. This has produced a skew in our knowledge, with the conclusions mainly based on a few members of the Hypocreales such as *Beauveria* and *Metarhizium* species [8]. The present study is one of the first that has aimed to document a full set of fungi isolated from several lepidopteran species (see also [24,25]). The detected diversity should inspire further studies—in addition to considerable bionomic data to be gained, the deeper knowledge would allow us to address the thus far little understood ecological role of pathogenic fungi in natural insect populations.

## Figures and Tables

**Table 1:** Species of fungi isolated from lepidopteran hosts (the field experiment data in brackets). UNITE species hypothesis (SH) codes are presented to facilitate communication on detected fungi [22].

Order/Family	Species	SH DOI *	Hosts **
**Hypocreales**/Cordycipitaceae	*Akanthomyces muscarius* (Petch) Spatafora, Kepler & B. Shrestha	SH1886969.08FU	EA 8 CP 4(+25) HP 1
	*Cordyceps bifusispora* O.E. Erikss.	SH1887323.08FU	CP (2) EA 1
	*Cordyceps farinosa* (Holmsk.) Kepler, B. Shrestha & Spatafora	SH1524463.08FU	EA 29 CP 6(+10) HP 7 UP 1
	*Lecanicillium praecognitum* Gorczak & Kisło	SH1524455.08FU	CC 1 EA 4
	*Simplicillium aogashimaense* Nonaka, Kaifuch i & Masuma	SH1988378.08FU	EA 1
	*Simplicillium lanosoniveum* (J.F.H Beyma) Zare & W. Gams	SH1988383.08FU	OG 1 HP 1
Clavicipitaceae	*Metapochonia bulbillosa* (W.Gams & Malla) Kepler, S.A.Rehner & Humber	SH1930500.08FU	CP 1(+2) EA 1
Hypocreaceae	*Trichoderma* cf. *aethiopicum* Mullaw, C.P. Kubicek & Samuels	SH1568015.08FU	CP (7)
	*Trichoderma koningii* Oudem.	SH2303517.08FU	CP 1 EA 1
	*Trichoderma trixiae/virilente/viridescens*	SH2303501.08FU	CP 1
	*Trichoderma viride* Pers.	SH2303512.08FU	EA 1
Nectriaceae	*Mariannaea camptospora* Samson	SH1506679.08FU	EA 1
	*Fusarium* cf. *sporotrichioides* Sherb.	SH2456045.08FU	EA 1 OG 1
	*Fusarium solani* species complex	SH2228332.08FU	HP 1
	*Fusarium tricinctum* (Corda) Sacc.	SH1919083.08FU	EA 2 HP 1 CP (5)
Tilachlidiaceae	*Tilachlidium brachiatum* (Batsch) Petch	SH1513367.08FU	CC 1 EA 2
**Eurotiales**/Aspergillaceae	*Penicillium thomii* Maire	SH2189918.08FU	EA 1 CP (12)
	*Penicillium* sp.	SH2283940.08FU	CP 1 EA 4 UP 1
	*Penicillium paczoskii* K.W. Zaleski	SH2189912.08FU	CP 1
**Dothideales**/Saccotheciaceae	*Aureobasidium pullulans* (de Bary) G. Arnaud	SH1872652.08FU	EA 2
**Capnodiales**/Cladosporiaceae	*Cladosporium delicatulum* Link	SH2320203.08FU	HP 1
**Mucorales**/Mucoraceae	*Mucor hiemalis* Wehmer	SH1989679.08FU	CP (17)
	*Mucor plumbeus* Bonord.	-	CP (25)
**Mortierellales**/Mortierellaceae	*Mortierella humilis* Linnem. ex W. Gams	SH2444324.08FU	EA 4 CP (1)
**Umbelopsidales**/Umbelopsidaceae	*Umbelopsis* sp.	-	EA 1

* DOI = digital object identifier, SH DOI is displayed here as a short code; ** insect hosts abbreviated as EA—*Ematurga atomaria*, CP—*Cabera pusaria*, HP—*Hypomecis punctinalis*, CC—*Chiasmia clathrata*, OP—*Orthosia gothica*, UP—unidentified pupa.

**Table 2:** Incidence of fungal infection in lab reared moths in different years.

Host Species	Year	Pupae	Fungal Prevalence %
*Ematurga atomaria*	2014	383	2.3
	2015	327	5.2
	2016	554	6
*Cabera pusaria*	2014	179	0
	2015	286	3.1
	2016	13	53.8
*Chiasmia clathrata*	2014	409	0.5
*Orthosia gothica*	2014	462	0.4
*Hypomecis punctinalis*	2015	360	1.7
	2016	5	60

**Table 3:** Demographic parameters of *C. pusaria* in the field experiment. Total sample size is shown in brackets.

Year	Survived until Pupation	Pupal Mortality	
		Fungi	Cause Unknown
2016	55.7% (140)	13.3% (45)	86.7% (45)
2017	29.9% (728)	55.5% (146)	44.5% (146)
